# Overview of Cochrane Systematic Reviews for Rehabilitation Interventions in Individuals with Upper Limb Fractures: A Mapping Synthesis

**DOI:** 10.3390/medicina60030469

**Published:** 2024-03-12

**Authors:** Sara Liguori, Antimo Moretti, Giuseppe Toro, Chiara Arienti, Michele Patrini, Carlotte Kiekens, Stefano Negrini, Giovanni Iolascon, Francesca Gimigliano

**Affiliations:** 1 Department of Medical and Surgical Specialties and Dentistry, University of Campania “Luigi Vanvitelli”, 80138 Naples, Italy; sara.liguori@unicampania.it (S.L.); antimo.moretti@unicampania.it (A.M.); giovanni.iolascon@unicampania.it (G.I.); 2 Department of Mental and Physical Health and Preventive Medicine, University of Campania “Luigi Vanvitelli”, 80138 Naples, Italy; francescagimigliano@gmail.com; 3 IRCCS Fondazione Don Carlo Gnocchi, 20148 Milan, Italy; carienti@dongnocchi.it; 4 IRCCS Istituto Ortopedico Galeazzi, 20161 Milan, Italy; mpatrini@dongnocchi.it (M.P.); carlotte.kiekens@isico.it (C.K.); stefano.negrini@unimi.it (S.N.); 5 Department of Biomedical, Surgical and Dental Sciences, Università “La Statale”, 20122 Milan, Italy

**Keywords:** fracture, rehabilitation, systematic review as topic, radius, elbow, humerus, evidence

## Abstract

*Background and Objectives*. This overview of Cochrane systematic reviews (CSRs) reports on current evidence on the effectiveness of rehabilitation interventions for persons with upper limb fractures (ULFs), and the quality of the evidence. *Materials and Methods*. Following the inclusion criteria defined by the World Health Organization, all CSRs tagged in the Cochrane Rehabilitation database that were relevant for persons with ULFs were included. A mapping synthesis was used to group outcomes and comparisons of included CSRs, indicating the effect of rehabilitation interventions and the certainty of evidence. *Results*. A total of three CSRs were included in the evidence map. The certainty of evidence was judged as low to very low. Early occupational and hand therapy, cyclic pneumatic soft tissue compression, and cross-education, when started during immobilization, may improve grip strength and wrist range of motion, with results maintained up to 12 weeks from the cast removal, compared to no intervention. Approaches such as occupational therapy and passive mobilisation, started post-immobilization, are probably safe in terms of secondary complications. However, the overall evidence of rehabilitative interventions related to proximal humeral fractures has been judged insufficient for all the outcomes considered. A paucity of primary studies and CSRs for elbow fractures was noted. *Conclusions*. This overview provided the effect and the certainty of evidence of rehabilitation interventions available after ULFs using a mapping synthesis. To date, there is a need to further the effectiveness and safety of these interventions for persons with ULFs, improving methodological quality of the research in the field.

## 1. Introduction

In 2017, the World Health Organization (WHO) launched the “Rehabilitation 2030: a call for action” initiative to improve rehabilitation globally as an essential component of integrated health services [1]. As part of this framework, the WHO Rehabilitation Programme is developing a Package of Interventions for Rehabilitation (PIR) for 20 health conditions to support ministries of health in integrating rehabilitation services into health systems [2]. These packages aim to cover the global needs for rehabilitation services which should be included as an essential part of universal health coverage to reach more people in need [3]. The development of the PIR takes a stepwise approach [2]: (1) selection of health conditions, (2) identification of interventions and best evidence, (3) selection of evidence-based interventions and their delivery platforms, (4) description of resource requirements, (5) peer review of the package, and (6) production of the alpha version of the PIR. The second step requires the identification of high certainty of evidence related to the effectiveness of interventions and has been named “Best Evidence for Rehabilitation” (be4rehab).

In total, 1 of the 20 selected health conditions is fractures. Fractures are a worldwide public health emergency occurring in individuals of all ages. Data from the Global Burden of Diseases, Injuries, and Risk Factors Study (GBD) 2019 framework, show that in 2019, there were 178 million new fractures [4]. Cieza et al. reported that fractures are the second most prevalent worldwide cause of the need for rehabilitation, with 436 million people living with a fracture for 26 million years of life lived with a disability [3]. Fracture incidence has constantly been rising for the past 30 years, particularly in the elderly female population [5]. Upper limb fractures (ULFs), including humerus, radius, and ulna, are reported among the top anatomical sites in terms of new fractures [4].

**Figure 1:**
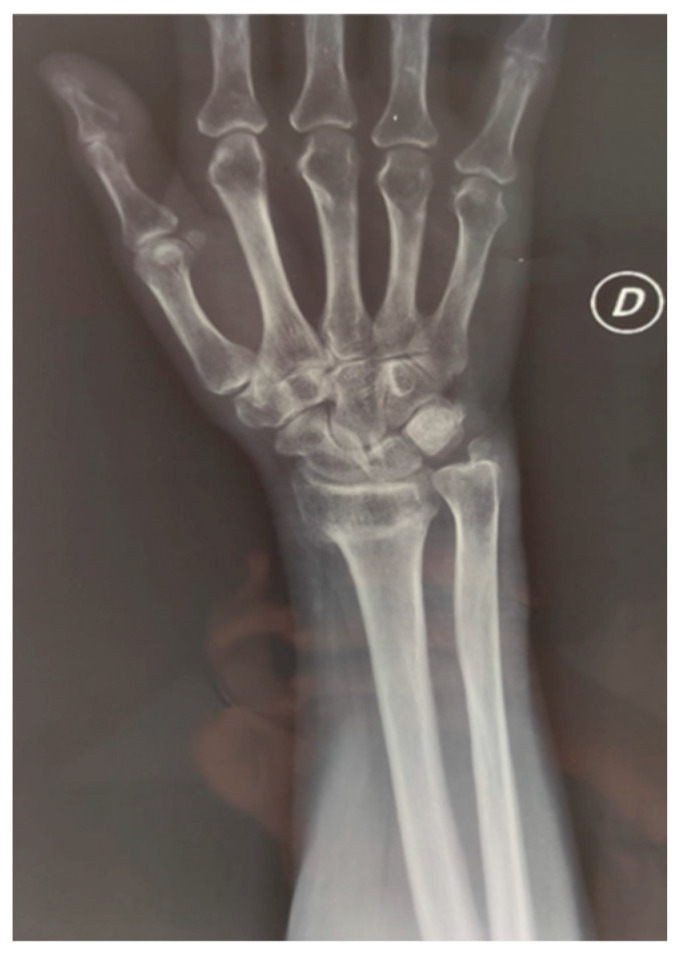
A classic finding of a distal radius fracture depicted by X-ray imaging.

The management of ULFs varies according to the severity of the fracture and the anatomical site [6,7] (Figure 1); moreover, modern imaging techniques like spectral computed tomography can support the detecting of fractures in doubtful cases, guiding the approach strategies [8]. For radius and/or ulna fractures, the conservative approach is generally preferred, as surgery is considered expensive and at higher risk for major complications [9]. More prompt and aggressive treatment is generally suggested for humerus fractures, as they present a high risk of delayed union or non-union [10]. Rehabilitation after ULFs is an important health strategy to optimize patients’ functioning. However, recommendations are lacking [11,12]. Considering that existing clinical practice guidelines on fractures are not focused on rehabilitative interventions, we examined systematic reviews of randomised controlled trials, widely recognised as the best form of evidence to inform treatment decisions [13]. Cochrane Systematic Reviews (CSRs) are the reference standard among systematic reviews for their rigorous methodology [14]. Cochrane Rehabilitation was requested by the WHO to search for evidence on rehabilitation interventions for the selected health conditions in the CSRs to be used as a starting point for PIR development.

This overview aims to describe the Cochrane evidence on rehabilitation interventions for persons with ULFs. The results provide an overall description of all the available Cochrane evidence in the field through the production of an evidence map [15].

## 2. Materials and Methods

In previous publications, we detailed the methodology [16] that follows what was established and published by the WHO Rehabilitation Programme and Cochrane Rehabilitation [2] under the guidance of the WHO’s guideline review committee. We performed an overview of CSRs reported following the Preferred Reporting Items for Systematic Reviews and Meta-analysis (PRISMA 2020 statement) [17] and registered on OSF Registries (https://doi.org/10.17605/OSF.IO/9QKYB (accessed on 1 December 2023)).

### 2.1. Search Strategy

**Figure 2:**
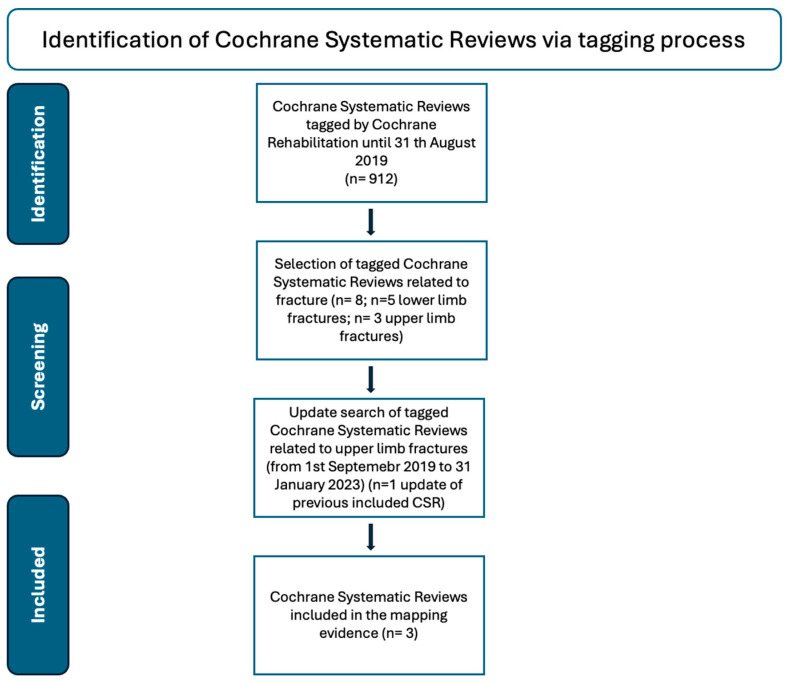
Flow chart displaying the tagging process of Cochrane systematic review.

The search strategy was developed by WHO in collaboration with Cochrane Rehabilitation. In a first search, the Cochrane Rehabilitation team included all the CSRs tagged by Cochrane Rehabilitation on fractures, from the inception of the Cochrane Library in 1996 to 31 August 2019 [18]. The search strings were composed of terms defining the “health condition” and, in this case, “fracture”, and “rehabilitation”. The search for the WHO was conducted in August 2019, and the data extracted were communicated to the WHO for the PIR development. Then, to move beyond the constraints of the rapid overview of reviews, as required by the WHO, we updated the research in the complete Cochrane Library to January 2023. For this overview, we selected the CSRs on ULFs (Figure 2).

### 2.2. Assessment of Methodological Quality of Included Reviews

To assess the methodological quality of the included CSRs, we used the 16-item AMSTAR 2 (A MeaSurement Tool to Assess systematic Reviews) [19]. According to WHO methodology [3], we specifically focused on seven items of AMSTAR 2. Two independent assessors (MP, SL) applied this instrument to all included CSRs, with any disagreement resolved through discussion with a third assessor (FG).

### 2.3. Data Extraction and Quality of Evidence Appraisal

As for previously published paper [16], we extracted data about each reported outcome related to an intervention from Table of Findings of each CSR. We extracted GRADE judgments within the CSRs when reported; otherwise, we judged the quality of evidence for the primary outcome, using the standard GRADE approach [20,21]. This process required the retrieval of the original primary studies included in each CSR.

### 2.4. Summarizing Evidence within a Map

Extracted data were summarized using tabular features identified as “mapping synthesis”, integrating outcomes and rehabilitation interventions to provide the grouping of outcomes and comparison of CSRs included and to give immediate information about the certainty of evidence (very low, low, moderate, and high) and effects (no effect, favour intervention, favour control).

## 3. Results

In total, 248 CSRs were tagged on the 20 selected health conditions identified by the PIR from the past 10 years; 3 of them were related to upper limb fractures.

**Figure 3.1:**
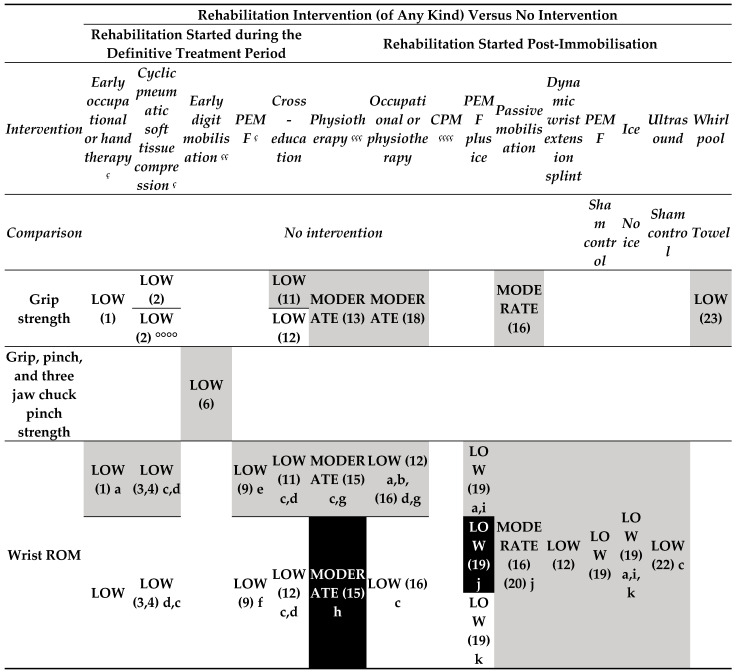

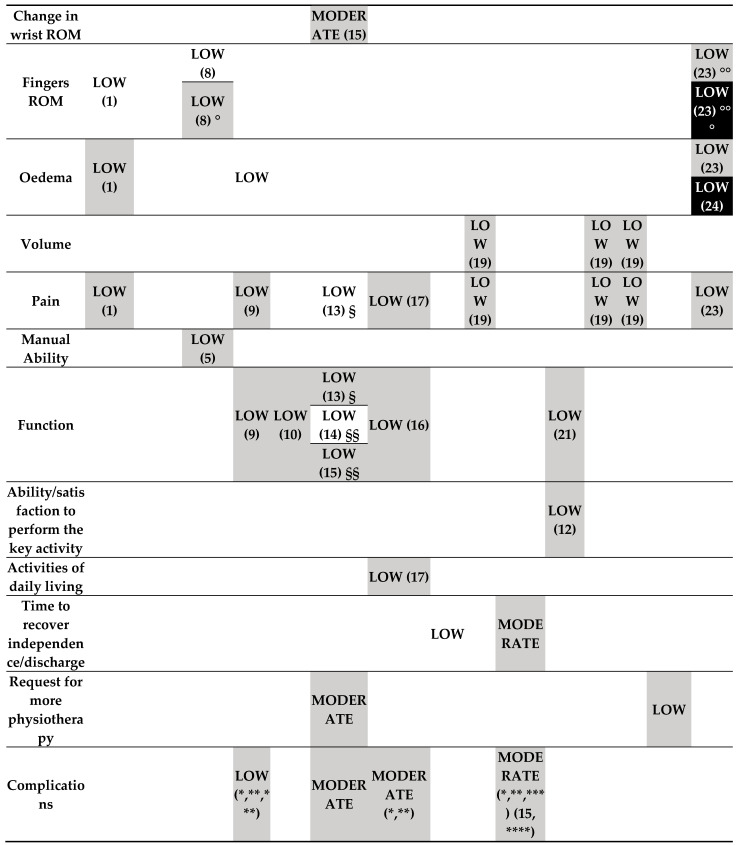
Evidence map for interventions in persons with distal radius fractures. **Map colours**: white: favour intervention; black: favour comparison; light grey—UPPERCASE: no effect; dark grey—lowercase: not estimable lower. Abbreviations: PEMF: pulsed electromagnetic field; CPM: continuous passive motion; ROM: range of motion; CRPS type 1: complex regional pain syndrome type 1; PRWE: Patient-Rated Wrist Evaluation; QuickDASH: Disabilities of the Arm, Shoulder, and Hand Quick Questionnaire; MCP: metacarpal phalange. ç: during immobilization; çç: during external fixation; ççç: one session for home exercises; çççç: post external fixation; 1: 4 weeks post-immobilisation; 2: 6 and 10 weeks post-immobilisation; 3: 6 weeks post-immobilisation; 4: 10 weeks post-immobilisation; 5: 3, 7, and 12 weeks post-immobilisation; 6: 7 and 12 weeks post-immobilisation; 8: 12 weeks post-immobilisation; 9: 2 to 3 days after cast removal; 10: 9, 12 and 26 weeks; 11: 9 and 26 weeks; 12: 12 weeks; 13: 3 and 6 weeks; 14: 3 weeks; 15: 6 weeks; 16: 24 weeks; 17: 3 and 6 months; 18: 3, 6 and 9 months; 19: at day 5; 20: 4 weeks; 21: 8 and 12 weeks; 22: 8 weeks; 23: at end of treatment; 24 at end of each session. a: Flexion, pronation, and radial deviation; b: extension, supination, and ulnar deviation; c: flexion/extension; d: pronation/supination; e: pronation, and ulnar and radial deviation; f: flexion, extension, and supination; g: radial/ulnar deviation; h: pronation; i: supination; j: extension; k: ulnar deviation; *: CRPS 1 (symptoms); **: median nerve compression; ***: finger stiffness; ****: malunion; §: PRWE; §§: Quick DASH; °: thumb; °°: Thumb, index, ring, and little fingers MCP ROM in flexion; °°°: Long finger MCP ROM in flexion; °°°°: pinch strength.

**Figure 3.2:**
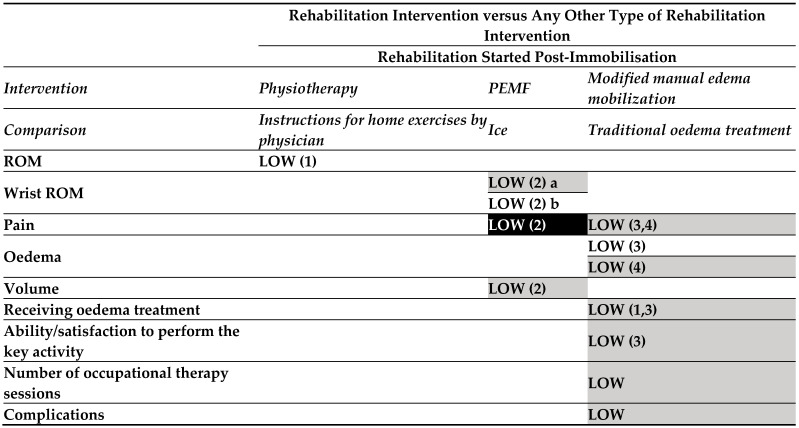
Evidence map for interventions in persons with distal radius fractures. **Map colours**: white: favour intervention; black: favour comparison; light grey—UPPERCASE: no effect; dark grey—lowercase: not estimable. Abbreviations: PEMF: pulsed electromagnetic field; ROM: range of motion. 1: 6 weeks; 2: at day 5; 3: 9 weeks; 4: 26 weeks; a: flexion, pronation, supination, and radial and ulnar deviation; b: extension.

**Figure 3.3:**
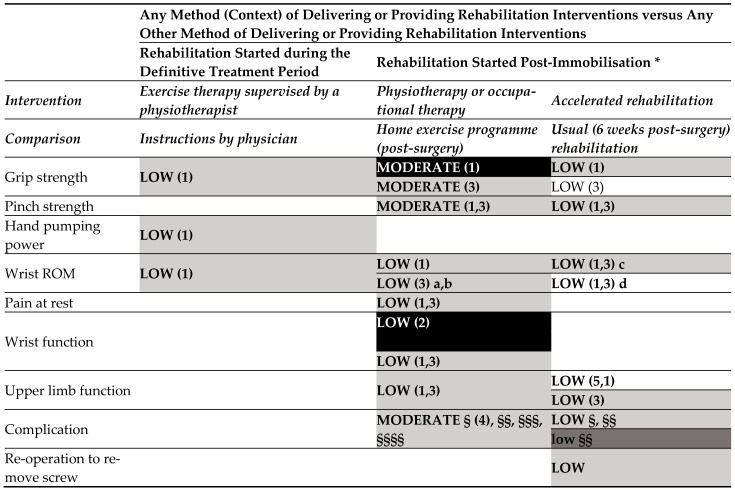
Evidence map for interventions in persons with distal radius fractures. **Map colours**: white: favour intervention; black: favour comparison; light grey—UPPERCASE: no effect; dark grey—lowercase: not estimable l. 1: 12 weeks; 2: 6 weeks; 3: 24 weeks; 4: 2–3 months post initial treatment; 5: 8 weeks; a: extension/flexion arc, extension, supination, and ulnar deviation; b: flexion, pronation, and radial deviation; c: extension, pronation, and supination; d: flexion; §: carpal tunnel release; §§: loss of alignment of lunar facet fragment; §§§: extensor pollicis longus tendon rupture; §§§§: implant removal for tendon irritation. * after volar plate fixation o. Abbreviations: ROM: range of motion.

**Figure 4:**
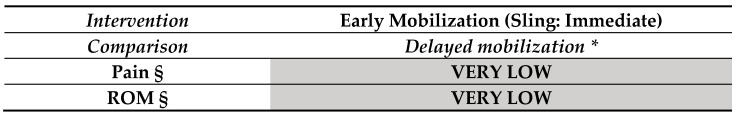
Evidence map for interventions in persons with elbow fractures. **Map colours**: white: favour intervention; black: favour comparison; light grey—UPPERCASE: no effect; dark grey—lowercase: not estimable. Abbreviations: POP: plaster of Paris cast; ROM: range of motion. * *(POP cast in flexion and/or extension: 2 weeks) § (mean follow-up 25 months, min 2 max 47)*.

**Figure 5:**
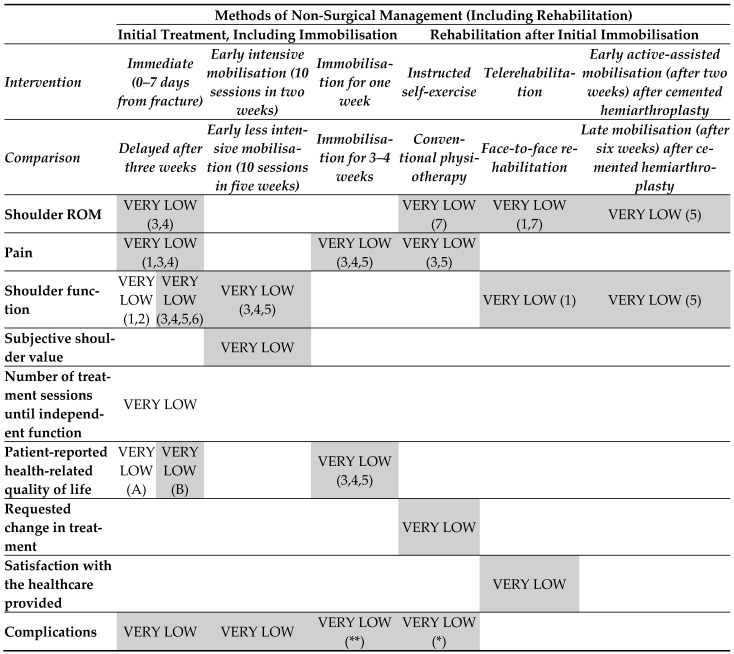
Evidence map for interventions in persons with proximal humeral fractures. **Map colours**: white: favour intervention; black: favour comparison; light grey—UPPERCASE: no effect; dark grey—lowercase: not estimable Abbreviations: ROM: range of motion; CRPS: complex regional pain syndrome 1: at 6/8 weeks; 2: at 16 weeks; 3: at 3 months; 4: at 6 months; 5 at 1 year; 6: at 2 years; 7: active A: pain/role limitation physical; B: other categories. *: frozen shoulder; **: CRPS TYPE 1.

The CSRs provide information on the effectiveness of rehabilitation interventions on several outcomes of proximal humeral [22], elbow [23], and radius [24]. Consequently, the evidence maps are limited to distal radial fractures, (Figure 3.1, Figure 3.2 and Figure 3.3) elbow (Figure 4), and proximal humeral fractures (Figure 5). CSRs’ characteristics are reported in Table S1.

The three available CSRs are judged as having high methodological quality by AMSTAR 2 tool, with the only negative aspect of not reporting information about funding sources (See Table S2).

### 3.1. Findings in Distal Radial Fractures

We collected effects of the comparisons divided by (i) rehabilitation intervention (started during the treatment period or post-immobilization) versus no intervention; (ii) one rehabilitation intervention (post-immobilization) versus another rehabilitation intervention; and (iii) any method of delivering or providing rehabilitation interventions (started during the treatment period or post-immobilization) versus any other method of delivering or providing rehabilitation interventions.

- Rehabilitation intervention versus no intervention (started during the treatment period) (Figure 3.1).
  o Low-certainty evidence

Occupational or other hand therapy at 4 weeks provided an effect on grip strength, wrist range of motion (ROM) (in extension, supination, and ulnar deviation), and finger mobility, while no effect was found in terms of other wrist movements, pain, and oedema.

Cyclic pneumatic soft tissue compression during immobilisation provided an affect on grip and pinch strength, and wrist ROM in pronation/supination (6 weeks post-immobilisation) and in flexion/extension (10 weeks post-immobilisation), while no effect was found in wrist ROM in flexion/extension (6 weeks post-immobilisation) and in pronation/supination (10 weeks post-immobilisation).

Early digit mobilization programme (during external fixation) provided an effect on finger mobility (12 weeks post-immobilisation).

Pulsed electromagnetic field (PEMF) (during cast immobilisation) provided no effect on pain and on wrist ROM, except for an effect in favour of intervention for wrist supination, flexion, extension, and oedema.

Intervention of cross education of the non-fractured hand was judged to provide no effect in terms of patient-reported wrist evaluation (PRWE) and on grip strength of the fractured hand and ROM wrist at 9 and 26 weeks, while an effect in favour of the intervention was found for the same grip strength and ROM wrist at 12 weeks.

- Rehabilitation intervention versus no intervention (started post-immobilisation) (Figure 3.1).

o Moderate-certainty of evidence

Comparing a single session of physiotherapy (advice and instructions for a home exercise programme) versus no intervention, no effect was found for grip strength, and ROM at 3 and 6 weeks (except for pronation in favour of the intervention at 6 weeks).

No effect was found for occupational or physiotherapy post-immobilization on grip strength of both affected (at 3, 6, and 9 months) and unaffected side (at 3 and 6 months) and in terms of complications (as Complex Regional Pain Syndrome-1 (CRPS-1) or carpal tunnel syndrome (CTS)).

o Low-certainty evidence

For the comparison of single session of physiotherapy (advice and instructions for a home exercise programme) this intervention provided effect in terms of PRWE on pain up to 6 weeks as well as functional outcomes like general QuickDASH at 3 weeks, while no clinically important between-group differences were found for each subitems of QuickDASH at 3 and 6 weeks. No effect was found for occupational or physiotherapy post-immobilisation on PRWE and ADLs (at 3 and 6 months), grip strength of both affected (at 3, 6, and 9 months) and unaffected side (at 3 and 6 months), pain (at 3 and 6 months), ROM (at 24 weeks), except for an effect in favour of intervention for flexion-extension ROM at 24 weeks. PEMF (post- immobilisation) was judged to provide no effect on pain and wrist ROM (at day 5) compared to sham control as well as for PEMF plus ice compared to no intervention, except for wrist ROM extension (in favour of control) and ulnar deviation (in favour of intervention). Similarly, no effect was found for ice application (post-immobilization) compared to no ice on pain and wrist ROM (at day 5) except for the degrees of wrist extension (in favour of intervention) and for passive mobilization in terms of grip strength, ROM, and complications (as CTS, finger stiffness CRPS-1 or malunion) at 6 weeks.

No effect on loss of wrist motion (>30%) was provided for low frequency, long-wave ultrasound (post- immobilisation) compared to sham control, as well as for whirlpool compared to towel in terms of grip strength, pain, or forearm and wrist ROM; whirlpool group showed statistically significantly worse scores on finger flexion and higher oedema.

Comparing dynamic splint to control group showed no statistically or clinically significant differences in the PRWE results at the end of the 8-week treatment or 1 month subsequently using the Canadian Occupational Performance Measure (COPM) and ROM at 12 weeks.

o Very-low-certainty evidence

It is uncertain if occupational therapy (OT) plus continuous passive motion provides an effect on time to achieve independent status compared to no intervention.

- One rehabilitation intervention (post-immobilization) versus another rehabilitation intervention (Figure 3.2).
  o Low-certainty evidence

From the comparison between physiotherapy and instructions for home exercises given by orthopaedic surgeons an effect in favour of the intervention group was found for wrist extension.

PEMF compared to ice was judged to provide no effect on volume and wrist ROM (at day 5), except for pain reduction and wrist extension in favour of control and intervention, respectively.

No effect was found comparing modified “manual oedema mobilization” (MEM) versus “traditional” oedema treatment in terms of COPM, pain “at rest” and “when active” (at 9 and 26 weeks), number of OT sessions, or timing of treatment (after 6 or 9 weeks), while an effect in favour of MEM was found in terms of oedema reduction after 9 weeks (not at 6 months).

- Any method of delivering or providing rehabilitation interventions versus any other method of delivering or providing rehabilitation interventions (started during the definitive treatment period) (Figure 3.3).
  o Low-certainty evidence

No effect on strength and ROM was found among exercise therapy supervised by a physiotherapist versus instructions for the same exercises given by an orthopaedic surgeon.

- Any method of delivering or providing rehabilitation interventions versus any other method of delivering or providing rehabilitation interventions (started post-immobilisation).

o Moderate-certainty of evidence

Physiotherapy or OT, compared to home exercise programme, were judged to provide no effect on complications (CTS, loss of alignment, extensor pollicis longus tendon rupture, implant removal for tendon irritation), pinch and grip strength at 3 and 6 months, and grip strength was better at 3 months in the controls.

o Low-certainty evidence

No difference in the effect was found on PRWE, and functional outcomes as DASH and Mayo score between physiotherapy or OT versus a home exercise programme post-surgery as well as on ROM at 3 and 6 months (except for extension-flexion arc, supination, extension, and ulnar deviation, all in favour of control).

Accelerated rehabilitation (started at 2 weeks after volar plate fixation) was judged to provide effect on DASH at 8 and 12 weeks (no effect at 6 months), while no effect was found on pinch strength at 12 weeks and 6 months, on grip strength at 12 weeks (effect in favour if intervention at 6 months), on ROM at 12 weeks and at 6 months (effect in favour of intervention on flexion ROM at 12 weeks and at 6 months), and on complications versus usual rehabilitation (started at 6 weeks) after volar plate fixation.

### 3.2. Findings in Elbow Fractures (Figure 4)

o Very-low-certainty evidence

All comparisons about early mobilization (immediate) were judged to provide very-low-certainty evidence on the effect on pain and ROM versus delayed mobilization (with a plaster of Paris cast—POP for 2 weeks) in either flexion and extension.

### 3.3. Findings in Proximal Humeral Fractures (Figure 5)

o Very-low-certainty evidence

All comparisons were judged to provide very-low-certainty evidence on the effect on shoulder function (at any time), patient-reported health-related quality of life (at any time), adverse events, pain (at any time), ROM, subjective shoulder value (at any time), requested change in treatment, and satisfaction with the healthcare provided.

## 4. Discussion

Our search of Cochrane evidence about rehabilitative interventions in the management of ULFs included just three CSRs with mostly low to very-low certainty of evidence [22,23,24]. The paucity of CSRs and the poor quality of evidence of the primary studies emphasize the need to further investigate the best rehabilitative approach post ULFs. The main outcomes of rehabilitation after ULFs are pain relief and regaining mobility, strength, and function [25,26]. For people with distal radius fractures, functional recovery is often fairly good after surgical or non-surgical treatment. However, rehabilitation could be beneficial to avoid common complaints referred to by patients including weakness, pain, and stiffness [27]. The interventions extracted from the CSRs and shown in our evidence map summarized evidence of rehabilitation started during plaster cast immobilisation or post-immobilization. Early occupational and hand therapy, cyclic pneumatic soft tissue compression, and cross-education, when started during immobilization, seems to improve grip strength and wrist ROM, with results maintained up to 12 weeks from the cast removal, compared to no intervention [26]. At the same time, interventions such as PEMF, ice, ultrasound, or whirlpool, compared to sham/no intervention or towel, started post-immobilization, may not improve the outcomes investigated and, in some cases, such as whirlpool versus towel, the comparison seems more beneficial than the intervention [27]. Approaches such as OT and passive mobilisation, started post-immobilization, are probably safe in terms of secondary complications (such as CRPS-1, median nerve compression, or finger stiffness) [26]. Concerning the method of delivering rehabilitative treatment after distal radius fracture, accelerated rehabilitation may be beneficial for function, ROM, and strength compared to rehabilitation provided 6 weeks post-surgery, while evidence of no effect was found for almost all other comparisons with, a certainty of evidence ranging from low to moderate [27]. Therefore, there is a lack of knowledge on which kind of rehabilitation and when/how long it is necessary to administer these interventions after distal radius fractures. This unmet need has also been highlighted by Gimigliano et al. in a previous systematic review examining the clinical practice guidelines (CPGs) including rehabilitative interventions for adults with fractures [11], where the two CPGs about radius fractures did not report recommendations on types, frequency, and timing of interventions, nor on post-acute rehabilitative approaches [28,29]. Another important aspect to consider is the poor adherence to rehabilitative treatment post distal radius fractures. In this scenario, several proposals of mobile serious games have been developed to guide recovery and improve adherence to treatment [30].

For what concerns elbow fractures, stiffness in extension is considered a common complication, making the timing of mobilization crucial to counteract this issue. However, the injury pattern and its management (surgical or not) could make the difference in terms of recovery of ROM and function due to the possible complications such as instability or displacement [31]. Our overview highlighted the scarcity of primary studies and CSRs on this topic. Indeed, the CSR included reported findings about one small trial, comparing early to delayed mobilization in patients with Mason type 1 and type 2 isolated radial head fracture [23,32]. However, this trial showed some related methodological issues, for example, to the reported findings and the detection of the outcomes analysed; moreover, the timing of follow-up established by the investigators does not match with the time from injury, leading to a huge range of follow-up in which the participants have been evaluated [32]. For that reason, the overall quality of evidence was judged as very low, leading to an unmet need related to the proper timing of mobilisation for this population. Despite the consensus about the necessity of high-quality rehabilitation post-elbow-fractures, there is a gap of knowledge on the state of the evidence-based practice rehabilitative approach for this condition [33]. Further research should face these issues, including other types of elbow fractures, and focus on additional outcomes, including upper limb function and quality of life.

Finally, the overall evidence of rehabilitative interventions related to proximal humeral fractures has been judged insufficient for all the outcomes considered. No evidence was available to be tested in the analysis for some comparisons, such as swimming pool treatment plus self-training versus self-training alone. Similarly to the CSR on elbow fractures, just small single trials have been found with serious imprecisions that suggest caution in interpreting the results [22]. The incidence of proximal humerus fractures is growing among elderly osteoporotic patients [34] where, due to lower functional needs, surgery is often avoided [35]. The common practice of non-surgical approaches in proximal humeral fractures (up to 80% of these fractures) includes the use of sling immobilization [36]. An early, progressive, and individual rehabilitation plan seems mandatory for this population [37]. Future research should be focused on well-designed studies on non-surgical treatments, preferring standard and validated outcome measures with adequate reporting of interventions, and including the potential applicability of the findings.

This overview described the best current evidence available in CSRs on rehabilitative interventions after ULFs. The decision to include only CSRs provided uniformity in the methodology applied and coherence in the evidence analysis, as suggested by Cochrane [14]. The use of the GRADE framework to rate the certainty of the evidence for all included CSRs made the methodological process homogeneous, applying a common, sensible, and transparent approach. The development of an evidence map synthesis could be worthwhile for an easy understanding of the current knowledge on this topic, facilitating its dissemination.

According to the established aims and to what WHO required, this overview reported only the GRADE evidence of current CSRs; therefore, a limitation is that it was not provided with an a priori grid, including all the possible outcomes and interventions in order to detect any evidence gap.

Another limitation is that the CSRs available on rehabilitative interventions for ULFs included small heterogeneous trials, with inadequate sample size and methodological shortcomings; for the one about elbow fractures, an update of the CSR is suggested, as it dates back more than ten years and includes a single trial published in 1994.

Future research is required to address priority questions, such as defining the provision, mode, and timing of rehabilitation after ULFs. Well-designed, powered, multicentre trials are required to implement the knowledge about the effectiveness and safety of rehabilitation after ULFs.

## 5. Conclusions

The evidence maps on rehabilitation following distal radius fractures suggest that interventions initiated during the treatment period showed limited low-quality evidence in favour of the interventions, while interventions that started post-immobilization showed moderate-certainty evidence for no effect. Additionally, comparisons between different rehabilitation interventions revealed that physiotherapy was more effective than home exercises for wrist extension, and MEM was more effective than traditional oedema treatment for oedema reduction after 9 weeks. However, there was no clear advantage observed when comparing different modes of delivery or provision of rehabilitation interventions. Furthermore, the effectiveness of rehabilitative interventions after elbow and proximal humeral fractures remains uncertain.

## Data Availability

Data will be available upon reasonable request.

## Supplementary Materials

The following supporting information can be downloaded at: https://www.mdpi.com/article/10.3390/medicina60030469/s1; Table S1. Characteristics of the studies included; Table S2. AMSTAR 2 Quality Assessment of Cochrane Systematic Reviews.
