# Supplementary material for: Overview of Cochrane Systematic Reviews for Rehabilitation Interventions in Individuals with Upper Limb Fractures: A Mapping Synthesis
Source: Medicina (Kaunas). 2024 Mar 12;60(3):469. doi: 10.3390/medicina60030469 (PMC10971878; doi:10.3390/medicina60030469)
Supplement: Supplementary file 1 [file medicina-60-00469-s001.zip › medicina-2811293-supplementary-table S1.docx]

| Authors | Title | Total N° included studies (N° participants) | Population | Setting | Intervention | Control | Outcome | Outcome Measurements | Groups | N° studies (N°participants) | Effect | GRADE |
| --- | --- | --- | --- | --- | --- | --- | --- | --- | --- | --- | --- | --- |
| Harding et al., 2011 | Early mobilisation for elbow fractures in adults | 1 (81) | People with elbow fractures | Hospital setting | Early mobilisation (sling: immediate) | Delayed mobilisation (POP cast in flexion or extension: 2 weeks) | Pain (mean follow-up 25 months, min 2 max 47) | Number of people with pain | both cast groups | 1 (81) | No effect | Very low |
|  |  |  |  |  |  |  |  |  | flexion cast group | 1 (58) |  |  |
|  |  |  |  |  |  |  |  |  | extension cast group | 1 (52) |  |  |
|  |  |  |  |  |  |  | ROM (mean follow-up 25 months, min 2 max 47) | Number of people with limited ROM | both cast groups | 1 (81) |  |  |
|  |  |  |  |  |  |  |  |  | flexion cast group | 1 (58) |  |  |
|  |  |  |  |  |  |  |  |  | extension cast group | 1 (52) |  |  |
| Handoll et al., 2015 | Rehabilitation for distal radial fractures in adults | 26 (1269) | People with distal radius fractures | Outpatient, inpatient, homepatient, NR | Early (during immobilisation) occupational or hand therapy | No intervention | Grip strength (4 weeks post-immobilisation) | Grip strength (kg) |  | 1 (17) | Favours intervention | Low |
|  |  |  |  |  |  |  | ROM (4 weeks post-immobilisation) | Degrees of pronation |  |  | No effect |  |
|  |  |  |  |  |  |  |  | Degrees of supination |  |  | Favours intervention |  |
|  |  |  |  |  |  |  |  | Degrees of flexion |  |  | No effect |  |
|  |  |  |  |  |  |  |  | Degrees of extension |  |  | Favours intervention |  |
|  |  |  |  |  |  |  |  | Degrees of radial deviation |  |  | No effect |  |
|  |  |  |  |  |  |  |  | Degrees of ulnar deviation |  |  | Favours intervention |  |
|  |  |  |  |  |  |  | Oedema (4 weeks post-immobilisation) | Oedema (mL) |  |  | No effect |  |
|  |  |  |  |  |  |  | Pain (4 weeks post-immobilisation) | Any pain at rest |  |  |  |  |
|  |  |  |  |  |  |  | Finger ROM (4 weeks post-immobilisation) | Thumb opposition (Kapandji score) |  |  | Favours intervention |  |
|  |  |  |  |  |  |  |  | Index finger (total active motion) (degrees) |  |  |  |  |
|  |  |  |  |  |  |  |  | Middle finger (total active motion) (degrees) |  |  |  |  |
|  |  |  |  |  |  |  |  | Ring finger (total active motion) (degrees) |  |  |  |  |
|  |  |  |  |  |  |  |  | Little Index finger (total active motion) (degrees) |  |  |  |  |
|  |  |  |  |  |  |  | Complications | CRPS-1 |  | (57) | Not estimable |  |
|  |  |  |  |  |  |  |  | Median nerve compression |  | 1 (17) |  |  |
|  |  |  |  |  |  |  |  | Ulnar nerve compression |  |  |  |  |
|  |  |  |  |  |  |  |  | Tendon rupture |  |  |  |  |
|  |  |  |  |  | Cyclic pneumatic soft tissue compression during immobilisation | No intervention | Grip strength | kg | 6 weeks post-immobilisation | 1 (19) | Favours intervention | Low |
|  |  |  |  |  |  |  |  |  | 10 weeks post-immobilisation |  |  |  |
|  |  |  |  |  |  |  | Pinch strength |  | 6 weeks post-immobilisation |  |  |  |
|  |  |  |  |  |  |  |  |  | 10 weeks post-immobilisation |  |  |  |
|  |  |  |  |  |  |  | ROM | Degrees of flexion/extension | 6 weeks post-immobilisation |  | No effect |  |
|  |  |  |  |  |  |  |  |  | 10 weeks post-immobilisation |  | Favours intervention |  |
|  |  |  |  |  |  |  |  | Degrees of pronation/supination | 6 weeks post-immobilisation |  |  |  |
|  |  |  |  |  |  |  |  |  | 10 weeks post-immobilisation |  | No effect |  |
|  |  |  |  |  | Early (during external fixation) digit mobilisation | No intervention | Manual Ability | MAM-36 - Taiwan version 45 questions | 3 weeks post-immobilisation | 1 (22) |  |  |
|  |  |  |  |  |  |  |  |  | 7 weeks post-immobilisation |  |  |  |
|  |  |  |  |  |  |  |  |  | 12 weeks post-immobilisation |  |  |  |
|  |  |  |  |  |  |  | Grip strength) | (% unaffected side) | 7 weeks post-immobilisation |  |  |  |
|  |  |  |  |  |  |  |  |  | 12 weeks post-immobilisation |  |  |  |
|  |  |  |  |  |  |  | Pinch strength |  | 7 weeks post-immobilisation |  |  |  |
|  |  |  |  |  |  |  |  |  | 12 weeks post-immobilisation |  |  |  |
|  |  |  |  |  |  |  | Three jaw chuck pinch strength |  | 7 weeks post-immobilisation |  |  |  |
|  |  |  |  |  |  |  |  |  | 12 weeks post-immobilisation |  |  |  |
|  |  |  |  |  |  |  | Finger ROM (12 weeks post-immobilisation) | Finger workspace (% unaffected side) |  |  | Favours intervention | Low |
|  |  |  |  |  |  |  |  | Thumb workspace (% unaffected side) |  |  | No effect |  |
|  |  |  |  |  | PEMF (during cast immobilisation) | No intervention | Pain (2 to 3 days after cast removal) | PRWE pain score |  | 1 (60) |  |  |
|  |  |  |  |  |  |  | Function (2 to 3 days after cast removal) | PRWE function score |  |  |  |  |
|  |  |  |  |  |  |  | ROM (2 to 3 days after cast removal) | Degrees of pronation |  |  |  |  |
|  |  |  |  |  |  |  |  | Degrees of supination |  |  | Favours intervention |  |
|  |  |  |  |  |  |  |  | Degrees of flexion |  |  |  |  |
|  |  |  |  |  |  |  |  | Degrees of extension |  |  |  |  |
|  |  |  |  |  |  |  |  | Degrees of radial deviation |  |  | No effect | Low |
|  |  |  |  |  |  |  |  | Degrees of ulnar deviation |  |  |  |  |
|  |  |  |  |  |  |  | Hand oedema | Difference between hands in circumference (mm) |  |  | Favours intervention |  |
|  |  |  |  |  |  |  | Complications | Total complications |  |  | No effect |  |
|  |  |  |  |  |  |  |  | CRPS 1 (symptoms) |  |  |  |  |
|  |  |  |  |  |  |  |  | Median nerve compression |  |  |  |  |
|  |  |  |  |  |  |  |  | Finger stiffness |  |  |  |  |
|  |  |  |  |  |  |  |  | Adverse effect of PEMF |  |  | Not estimable |  |
|  |  |  |  |  | Cross-education (strengthening exercises for opposite hand) | No intervention | Wrist Disability | PRWE | 9 weeks | 1 (39) | No effect | Low |
|  |  |  |  |  |  |  |  |  | 12 weeks |  |  |  |
|  |  |  |  |  |  |  |  |  | 26 weeks |  |  |  |
|  |  |  |  |  |  |  | Grip strength | kg | (9 weeks) |  |  |  |
|  |  |  |  |  |  |  |  |  | (12 weeks) |  | Favours intervention |  |
|  |  |  |  |  |  |  |  |  | (26 weeks) |  | No effect |  |
|  |  |  |  |  |  |  | ROM | Degrees of supination /pronation | 9 weeks |  |  |  |
|  |  |  |  |  |  |  |  |  | 12 weeks |  | Favours intervention |  |
|  |  |  |  |  |  |  |  |  | 26 weeks |  | No effect |  |
|  |  |  |  |  |  |  |  | Degrees of flexion/extension | 9 weeks |  |  |  |
|  |  |  |  |  |  |  |  |  | 12 weeks |  | Favours intervention |  |
|  |  |  |  |  |  |  |  |  | 26 weeks |  | No effect |  |
|  |  |  |  |  | Physiotherapy (one session for home exercises) | No intervention | Pain | PRWE pain score | 3 weeks | 1 (48) | Favours intervention |  |
|  |  |  |  |  |  |  |  |  | 6 weeks | 1 (47) |  |  |
|  |  |  |  |  |  |  | Function | PRWE function score | 3 weeks | 1 (48) | No effect |  |
|  |  |  |  |  |  |  |  |  | 6 weeks | 1 (47) |  |  |
|  |  |  |  |  |  |  | Disability (3 weeks) | QuickDASH (general) | 3 weeks | 1 (48) | Favours intervention |  |
|  |  |  |  |  |  |  |  |  | 6 weeks | 1 (47) | No effect |  |
|  |  |  |  |  |  |  | Work disability | QuickDASH (work) | 3 weeks | 1 (48) |  |  |
|  |  |  |  |  |  |  |  |  | 6 weeks | 1 (47) |  |  |
|  |  |  |  |  |  |  | Sports disability | QuickDASH (sports) | 3 weeks | 1 (48) |  |  |
|  |  |  |  |  |  |  |  |  | 6 weeks | 1 (47) |  |  |
|  |  |  |  |  |  |  | Grip strength | kg | 3 weeks | 1 (48) |  | Moderate |
|  |  |  |  |  |  |  |  |  | 6 weeks | 1 (47) |  |  |
|  |  |  |  |  |  |  | ROM (6 weeks) | Degrees of extension |  |  |  |  |
|  |  |  |  |  |  |  |  | Degrees of flexion |  |  |  |  |
|  |  |  |  |  |  |  |  | Degrees of radial deviation |  |  |  |  |
|  |  |  |  |  |  |  |  | Degrees of ulnar deviation |  |  |  |  |
|  |  |  |  |  |  |  |  | Degrees of pronation |  |  | Favours control |  |
|  |  |  |  |  |  |  |  | Degrees of supination |  |  | No effect |  |
|  |  |  |  |  |  |  | Change in ROM (6 weeks) | Degrees of extension |  |  |  |  |
|  |  |  |  |  |  |  |  | Degrees of flexion |  |  |  |  |
|  |  |  |  |  |  |  |  | Degrees of radial deviation |  |  |  |  |
|  |  |  |  |  |  |  |  | Degrees of ulnar deviation |  |  |  |  |
|  |  |  |  |  |  |  |  | Degrees of pronation |  |  |  |  |
|  |  |  |  |  |  |  |  | Degrees of supination |  |  |  |  |
|  |  |  |  |  |  |  | Complications |  |  | 1 (56) |  |  |
|  |  |  |  |  |  |  | Request for more physiotherapy |  |  |  |  |  |
|  |  |  |  |  | Post-immobilisation occupational or physiotherapy | No intervention | Wrist function (24 weeks) | PRWE |  | 1 (33) | No effect | Low |
|  |  |  |  |  |  |  | Pain (24 weeks) | PRWE pain score |  |  |  |  |
|  |  |  |  |  |  |  | Activity (24 weeks) | PRWE activity score |  |  |  |  |
|  |  |  |  |  |  |  | Disability (24 weeks) | PRWE disability score |  |  |  |  |
|  |  |  |  |  |  |  | Activities of daily living | Activities of daily living scores (% unaffected side) | Activities of daily living (3 months) | 1 (90) |  |  |
|  |  |  |  |  |  |  |  |  | Activities of daily living (6 months) | 1 (66) |  |  |
|  |  |  |  |  |  |  | Grip strength | kg | 3 months | 1 (30) | No effect | Moderate |
|  |  |  |  |  |  |  |  |  | 6 months | 1 (33) |  |  |
|  |  |  |  |  |  |  |  |  | 9 months | 1 (30) |  |  |
|  |  |  |  |  |  |  | Grip strength | % unaffected side | 3 months | 1 (90) |  |  |
|  |  |  |  |  |  |  |  |  | 6 months | 1 (66) |  |  |
|  |  |  |  |  |  |  | Pain | VAS | 3 months | 1 (90) |  | Low |
|  |  |  |  |  |  |  |  |  | 6 months | 1 (66) |  |  |
|  |  |  |  |  |  |  | ROM (6 months) | Degrees of flexion |  | 1 (33) |  |  |
|  |  |  |  |  |  |  |  | Degrees of extension |  |  |  |  |
|  |  |  |  |  |  |  | ROM | Pronation /supination (% unaffected side) | 3 months | 1 (90) |  |  |
|  |  |  |  |  |  |  |  |  | 6 months | 1 (66) |  |  |
|  |  |  |  |  |  |  |  | Flexion/extension (% unaffected side) | 3 months | 1 (90) |  |  |
|  |  |  |  |  |  |  |  |  | 6 months | 1 (66) | Favours intervention |  |
|  |  |  |  |  |  |  |  | Radial/ulnar deviation (% unaffected side) | 3 months | 1 (90) | No effect |  |
|  |  |  |  |  |  |  |  |  | 6 months | 1 (66) |  |  |
|  |  |  |  |  |  |  | N of treatments |  |  | 1 (41) | Favours control |  |
|  |  |  |  |  |  |  | Complications - CRPS-1 |  |  | 1 (98) | No effect | Moderate |
|  |  |  |  |  |  |  | Complications - Carpal tunnel syndrome |  |  | 1 (98) |  |  |
|  |  |  |  |  | CPM (post-external fixation) | No intervention | Time to recover independence | Time (weeks) |  | 1 (7) | Favours intervention |  |
|  |  |  |  |  | PEMF (post-immobilisation) | Sham control | Pain (at day 5) | VAS |  | 1 (83) | No effect | Low |
|  |  |  |  |  |  |  | Volume (at day 5) | mL |  |  |  |  |
|  |  |  |  |  |  |  | ROM (at day 5) | Degrees of pronation |  |  |  |  |
|  |  |  |  |  |  |  |  | Degrees of supination |  |  |  |  |
|  |  |  |  |  |  |  |  | Degrees of flexion |  |  |  |  |
|  |  |  |  |  |  |  |  | Degrees of extension |  |  |  |  |
|  |  |  |  |  |  |  |  | Degrees of radial deviation |  |  |  |  |
|  |  |  |  |  |  |  |  | Degrees of ulnar deviation |  |  |  |  |
|  |  |  |  |  | Ice (post-immobilisation) | No ice | Pain (at day 5) | VAS |  |  |  |  |
|  |  |  |  |  |  |  | Volume (at day 5) | mL |  |  |  |  |
|  |  |  |  |  |  |  | ROM (at day 5) | Degrees of pronation |  |  |  |  |
|  |  |  |  |  |  |  |  | Degrees of supination |  |  |  |  |
|  |  |  |  |  |  |  |  | Degrees of flexion |  |  |  |  |
|  |  |  |  |  |  |  |  | Degrees of extension |  |  | Favours control |  |
|  |  |  |  |  |  |  |  | Degrees of radial deviation |  |  | No effect |  |
|  |  |  |  |  |  |  |  | Degrees of ulnar deviation |  |  |  |  |
|  |  |  |  |  | PEMF plus ice (post-immobilisation) | No intervention | Pain (at day 5) | VAS |  | 1 (39) |  |  |
|  |  |  |  |  |  |  | Volume (at day 5) | mL |  |  |  |  |
|  |  |  |  |  |  |  | ROM (at day 5) | Degrees of pronation |  |  |  |  |
|  |  |  |  |  |  |  |  | Degrees of supination |  |  |  |  |
|  |  |  |  |  |  |  |  | Degrees of flexion |  |  |  |  |
|  |  |  |  |  |  |  |  | Degrees of extension |  |  | Favours control |  |
|  |  |  |  |  |  |  |  | Degrees of radial deviation |  |  | No effect |  |
|  |  |  |  |  |  |  |  | Degrees of ulnar deviation |  |  | Favours intervention |  |
|  |  |  |  |  | Passive mobilisation (post-immobilisation) | No intervention | Grip strength (6 weeks) | Grip strength (kg) |  |  | No effect | Moderate |
|  |  |  |  |  |  |  | ROM (6 weeks) | Degrees of pronation |  |  |  |  |
|  |  |  |  |  |  |  |  | Degrees of supination |  |  |  |  |
|  |  |  |  |  |  |  |  | Degrees of flexion |  |  |  |  |
|  |  |  |  |  |  |  |  | Degrees of extension |  |  |  |  |
|  |  |  |  |  |  |  |  | Degrees of radial deviation |  |  |  |  |
|  |  |  |  |  |  |  |  | Degrees of ulnar deviation |  |  |  |  |
|  |  |  |  |  |  |  |  | Degrees of web space angle |  |  |  |  |
|  |  |  |  |  |  |  | ROM (at discharge 4 weeks) | Degrees of wrist extension |  | 1 (30) |  |  |
|  |  |  |  |  |  |  | N of treatments |  |  | 2 (69) | na | na |
|  |  |  |  |  |  |  | Time to discharge | Time (days) |  | 1 (30) | No effect | Moderate |
|  |  |  |  |  |  |  | Complications at 6 weeks | Carpal tunnel syndrome |  | 1 (39) |  |  |
|  |  |  |  |  |  |  |  | Finger stiffness (continuing) |  |  |  |  |
|  |  |  |  |  |  |  |  | CRPS (continuing) |  |  |  |  |
|  |  |  |  |  |  |  |  | Malunion |  |  |  |  |
|  |  |  |  |  | Low frequency, long-wave ultrasound (post-immobilisation) | Sham control | ROM (8 weeks) | Greater than 30% loss of wrist flexion/extension |  | 1 (38)  1 (38) |  | Low |
|  |  |  |  |  |  |  | Referral for physiotherapy |  |  |  |  |  |
|  |  |  |  |  | Whirlpool (post-immobilisation) | Towel | Grip strength (end of treatment) | Grip strength (kg) |  | 1 (24) |  |  |
|  |  |  |  |  |  |  | Pain (end of treatment) | 0-5 scale |  |  |  |  |
|  |  |  |  |  |  |  | ROM (end of treatment) | Degrees of pronation |  |  |  |  |
|  |  |  |  |  |  |  |  | Degrees of supination |  |  |  |  |
|  |  |  |  |  |  |  |  | Degrees of flexion |  |  |  |  |
|  |  |  |  |  |  |  |  | Degrees of extension |  |  |  |  |
|  |  |  |  |  |  |  |  | Degrees of radial deviation |  |  |  |  |
|  |  |  |  |  |  |  |  | Degrees of ulnar deviation |  |  |  |  |
|  |  |  |  |  |  |  |  | Degrees of thumb MCP flexion |  |  |  |  |
|  |  |  |  |  |  |  |  | Degrees of index finger MCP flexion |  |  |  |  |
|  |  |  |  |  |  |  |  | Degrees of long finger MCP flexion |  |  | Favours control |  |
|  |  |  |  |  |  |  |  | Degrees of ring finger MCP flexion |  |  | No effect |  |
|  |  |  |  |  |  |  |  | Degrees of little finger MCP flexion |  |  |  |  |
|  |  |  |  |  |  |  | Oedema | ml | at end of each session |  | Favours control |  |
|  |  |  |  |  |  |  |  |  | at end of treatment |  | No effect |  |
|  |  |  |  |  | Dynamic wrist extension splint | No intervention | Wrist function | PRWE | 8 weeks | 1 (36) |  |  |
|  |  |  |  |  |  |  |  |  | 12 weeks | 1 (32) |  |  |
|  |  |  |  |  |  |  |  |  | 12 weeks, per protocol analysis |  |  |  |
|  |  |  |  |  |  |  | Ability to perform the key activity (12 weeks) | Canadian Occupational Performance Measure |  |  |  |  |
|  |  |  |  |  |  |  | Satisfaction with ability to perform key activity (12 weeks) |  |  |  |  |  |
|  |  |  |  |  |  |  | ROM (12 weeks) | Degrees of passive wrist extension |  |  |  |  |
|  |  |  |  |  |  |  |  | Degrees of active wrist extension |  |  |  |  |
|  |  |  |  |  |  |  |  | Degrees of active wrist flexion |  |  |  |  |
|  |  |  |  |  |  |  |  | Degrees of active radial deviation |  |  |  |  |
|  |  |  |  |  |  |  |  | Degrees of active ulnar deviation |  |  |  |  |
|  |  |  |  |  | Post-immobilisation physiotherapy | Instructions by physician | ROM (6 weeks) | Degrees of wrist extension |  | 1 (16) | Favours intervention |  |
|  |  |  |  |  | PEMF | Ice | Pain (at day 5) | VAS |  | 1(44) | Favours control |  |
|  |  |  |  |  |  |  | Volume (at day 5) | mL |  |  | No effect |  |
|  |  |  |  |  |  |  | ROM (at day 5) | Degrees of pronation |  |  |  |  |
|  |  |  |  |  |  |  |  | Degrees of supination |  |  |  |  |
|  |  |  |  |  |  |  |  | Degrees of flexion |  |  |  |  |
|  |  |  |  |  |  |  |  | Degrees of extension |  |  | Favours intervention |  |
|  |  |  |  |  |  |  |  | Degrees of radial deviation |  |  | No effect |  |
|  |  |  |  |  |  |  |  | Degrees of ulnar deviation |  |  |  |  |
|  |  |  |  |  | MEM | Traditional oedema treatment | Ability to perform the key activity (9 weeks) | Canadian Occupational Performance Measure |  | 1(28) |  |  |
|  |  |  |  |  |  |  | Satisfaction with ability to perform key activity (9 weeks) |  |  |  |  |  |
|  |  |  |  |  |  |  | Pain | VAS | at rest (9 weeks) | 1(29) |  |  |
|  |  |  |  |  |  |  |  |  | at rest (26 weeks) |  |  |  |
|  |  |  |  |  |  |  |  |  | when active (9 weeks) |  |  |  |
|  |  |  |  |  |  |  |  |  | when active (26 weeks) |  |  |  |
|  |  |  |  |  |  |  | Complications |  |  |  |  |  |
|  |  |  |  |  |  |  | Number of occupational therapy sessions |  |  |  |  |  |
|  |  |  |  |  |  |  | Receiving oedema treatment |  | after 6 weeks |  |  |  |
|  |  |  |  |  |  |  |  |  | after 9 weeks |  |  |  |
|  |  |  |  |  |  |  | Oedema | Volume difference between injured and non-injured side (mL) | 9 weeks |  | Favours intervention |  |
|  |  |  |  |  |  |  |  |  | 26 weeks |  | No effect |  |
|  |  |  |  |  | Supervised training by physiotherapist | Instructions by physician | Grip strength (12 weeks) | Grip strength (kg/cm2) |  | 1(96) |  |  |
|  |  |  |  |  |  |  | Hand pumping power (12 weeks) | Hand pumping power (mmHg) |  |  |  |  |
|  |  |  |  |  |  |  | ROM (12 weeks) | Degrees of pronation |  |  |  |  |
|  |  |  |  |  |  |  |  | Degrees of supination |  |  |  |  |
|  |  |  |  |  |  |  |  | Degrees of flexion |  |  |  |  |
|  |  |  |  |  |  |  |  | Degrees of extension |  |  |  |  |
|  |  |  |  |  |  |  |  | Degrees of radial deviation |  |  |  |  |
|  |  |  |  |  |  |  |  | Degrees of ulnar deviation |  |  |  |  |
|  |  |  |  |  | Physiotherapy/occupational therapy (post-surgery) | Home exercise programme (post-surgery) | Wrist function (6 weeks) | PRWE |  | 1(46) | Favours control |  |
|  |  |  |  |  |  |  | Disability | DASH | 3 months | 1(90) | No effect |  |
|  |  |  |  |  |  |  |  |  | 6 months | 1(76) |  |  |
|  |  |  |  |  |  |  | Wrist function | Mayo wrist score | 3 months | 1(90) |  |  |
|  |  |  |  |  |  |  |  |  | 6 months | 1(76) |  |  |
|  |  |  |  |  |  |  | Pain at rest | VAS | 3 months | 1(90) |  |  |
|  |  |  |  |  |  |  |  |  | 6 months | 1(76) |  |  |
|  |  |  |  |  |  |  | Grip strength | kg | 3 months | 1(90) | Favours control | Moderate |
|  |  |  |  |  |  |  |  |  | 6 months | 1(76) | No effect |  |
|  |  |  |  |  |  |  | Pinch strength |  | 3 months | 1(90) |  |  |
|  |  |  |  |  |  |  |  |  | 6 months | 1(76) |  |  |
|  |  |  |  |  |  |  | ROM | Degrees extension/flexion arc | 12 weeks | 1(90) |  | Low |
|  |  |  |  |  |  |  |  |  | 24 weeks | 1(76) | Favours control |  |
|  |  |  |  |  |  |  |  | Degrees of pronation | 12 weeks | 1(90) | No effect |  |
|  |  |  |  |  |  |  |  |  | 24 weeks | 1(76) |  |  |
|  |  |  |  |  |  |  |  | Degrees of supination | 12 weeks | 1(90) |  |  |
|  |  |  |  |  |  |  |  |  | 24 weeks | 1(76) | Favours control |  |
|  |  |  |  |  |  |  |  | Degrees of flexion | 12 weeks | 1(90) | No effect |  |
|  |  |  |  |  |  |  |  |  | 24 weeks | 1(76) |  |  |
|  |  |  |  |  |  |  |  | Degrees of extension | 12 weeks | 1(90) |  |  |
|  |  |  |  |  |  |  |  |  | 24 weeks | 1(76) | Favours control |  |
|  |  |  |  |  |  |  |  | Degrees of radial deviation | 12 weeks | 1(90) | No effect |  |
|  |  |  |  |  |  |  |  |  | 24 weeks | 1(76) |  |  |
|  |  |  |  |  |  |  |  | Degrees of ulnar deviation | 12 weeks | 1(90) |  |  |
|  |  |  |  |  |  |  |  |  | 24 weeks | 1(76) | Favours control |  |
|  |  |  |  |  |  |  | Complications | Carpal tunnel release (2-3 months post-initial treatment) |  | 1(94) | No effect | Moderate |
|  |  |  |  |  |  |  |  | Loss of alignment of lunar facet fragment |  |  |  |  |
|  |  |  |  |  |  |  |  | Extensor pollicis longus tendon rupture |  |  |  |  |
|  |  |  |  |  |  |  |  | Implant removal for tendon irritation |  |  |  |  |
|  |  |  |  |  | Accelerated (2 weeks post-surgery) rehabilitation | Usual (6 weeks post-surgery) rehabilitation | Disability | DASH | 8 weeks | 1(67) | Favours intervention | Low |
|  |  |  |  |  |  |  |  |  | 12 weeks |  |  |  |
|  |  |  |  |  |  |  |  |  | 24 weeks | 1(63) | No effect |  |
|  |  |  |  |  |  |  | Grip strength | lb | 12 weeks | 1(67) |  |  |
|  |  |  |  |  |  |  |  |  | 24 weeks | 1(63) | Favours intervention |  |
|  |  |  |  |  |  |  | Pinch strength |  | 12 weeks | 1(67) | No effect |  |
|  |  |  |  |  |  |  |  |  | 24 weeks | 1(63) |  |  |
|  |  |  |  |  |  |  | ROM (12 weeks) | Degrees of pronation | 12 weeks | 1(67) |  |  |
|  |  |  |  |  |  |  |  |  | 24 weeks | 1(63) |  |  |
|  |  |  |  |  |  |  |  | Degrees of supination | 12 weeks | 1(67) |  |  |
|  |  |  |  |  |  |  |  |  | 24 weeks | 1(63) |  |  |
|  |  |  |  |  |  |  |  | Degrees of flexion | 12 weeks | 1(67) | Favours intervention |  |
|  |  |  |  |  |  |  |  |  | 24 weeks | 1(63) |  |  |
|  |  |  |  |  |  |  |  | Degrees of extension | 12 weeks | 1(67) | No effect |  |
|  |  |  |  |  |  |  |  |  | 24 weeks | 1(63) |  |  |
|  |  |  |  |  |  |  | Complications | Carpal tunnel syndrome |  | 1(78) |  |  |
|  |  |  |  |  |  |  |  | Loss of alignment or non-union |  |  | Not estimable |  |
|  |  |  |  |  |  |  |  | Extensor pollicis longus tendon rupture |  |  | No effect | Low |
|  |  |  |  |  |  |  |  | Re-operation to remove screw |  |  |  |  |
| Handoll et al., 2022 | Interventions for treating proximal humeral fractures in adults | 47 (3179) | People with proximal humeral fractures | Mixed (home, clinic) | Immediate physiotherapy (gradual assisted movements of the upper limb) within one week of fracture | Delayed phys- iotherapy after three weeks of immobilisation in a collar and cuff sling | Shoulder function | Croft Shoulder Disability Questionnaire | at 1 years | 1 (86) | No effect | Very low |
|  |  |  |  |  |  |  |  |  | at 2 years |  |  |  |
|  |  |  |  |  |  |  |  |  | severity one/two years |  |  |  |
|  |  |  |  |  |  |  |  | Constant score (ratio of affected/unaffected arm) | at 8-16 weeks |  | Favours interventions |  |
|  |  |  |  |  |  |  |  |  | at 1 year |  | No effect |  |
|  |  |  |  |  |  |  | Number of treatment sessions (until independent function achieved) | NR |  |  | Favours intervention |  |
|  |  |  |  |  |  |  | Patient-reported health-related quality of life | SF-36-role limitation physical |  |  |  |  |
|  |  |  |  |  |  |  |  | SF-36-pain |  |  |  |  |
|  |  |  |  |  |  |  |  | SF-36-other categories |  |  | No effect |  |
|  |  |  |  |  |  |  | Adverse events | Frozen shoulder |  |  |  |  |
|  |  |  |  |  | Immobilisation in sling and body bandage for one week | Immobilisation in sling and body bandage for three weeks | Adverse events | CRPS type 1 |  | 1 (85) |  |  |
|  |  |  |  |  | Physiotherapy started within 3 days of fracture:  2-hour sessions supervised by a physiotherapist, 5 times a week. Then twiceaweek after 3 weeks | Physiotherapy started after 3 weeks of immo- bilisation in a sling:  2-hour sessions supervised by a physiotherapist, 4 times a week for 4 weeks | Pain | VAS at 3 months |  | 1 (74) |  |  |
|  |  |  |  |  |  |  |  | VAS at 6 weeks/at 6 months |  |  |  |  |
|  |  |  |  |  |  |  | ROM | Degrees, at 3-6 months |  |  |  |  |
|  |  |  |  |  |  |  | Adverse events | Treated (injection) subacromial impingement/Shoulder complications |  |  |  |  |
|  |  |  |  |  | Physiotherapy (pendulum move- ments) started immediately after diagnosis of injury | Physiotherapy delayed until three weeks | Shoulder function | QuickDASH score at 3-6 months |  | 1 (63) |  |  |
|  |  |  |  |  |  |  | Pain | Likert scale at 3-6 months |  |  |  |  |
|  |  |  |  |  |  |  | ROM | Degrees at 3-6 months |  |  |  |  |
|  |  |  |  |  | Immobilisation in sling for one week | Immobilisation in sling for four weeks | Patient-reported health-related quality of life | EQ-5D at 3 months |  | 1 (42) |  |  |
|  |  |  |  |  |  |  |  | EQ-5D at 6 months |  |  |  |  |
|  |  |  |  |  |  |  |  | EQ-5D at 12 months |  |  |  |  |
|  |  |  |  |  |  |  | Adverse events | Fracture displacement/ Shoulder complications/ fracture complications |  |  |  |  |
|  |  |  |  |  |  |  | Pain | VAS at 3-6-12 months |  |  |  |  |
|  |  |  |  |  | Early intensive mobilisation (10 sessions in two weeks) | Early less intensive mobilisation (10 sessions in five weeks) started one week after the fracture, during sling use | Subjective shoulder value | Subjective shoulder value at 3,6,12 months |  | 1 (80) |  |  |
|  |  |  |  |  |  |  | Adverse events | Fracture nonunion/ loss of reduction |  |  |  |  |
|  |  |  |  |  |  |  | Shoulder function | Constant score at 3 months |  |  |  |  |
|  |  |  |  |  |  |  |  | Constant score at 6-12 months |  |  | Favour control |  |
|  |  |  |  |  | Instructed self-exercise | Conventional physiotherapy | Adverse events | Frozen shoulder |  | 2 (62) | No effects |  |
|  |  |  |  |  |  |  | Neer's scores |  |  |  |  |  |
|  |  |  |  |  |  |  | Pain | Scale (0 to 8 points) at 1 year or severe or moderate pain at 3 months |  |  |  |  |
|  |  |  |  |  |  |  | Requested change in treatment |  |  |  |  |  |
|  |  |  |  |  |  |  | Active glenohumeral elevation | Degrees |  |  |  |  |
|  |  |  |  |  | Telerehabilitation | Face-to-face rehabilitation | Shoulder function at 8 weeks | QuickDASH score |  | 1 (30) |  |  |
|  |  |  |  |  |  |  |  | Constant score (ratio of affected/unaffected arm) |  |  |  |  |
|  |  |  |  |  |  |  | Active range of motion at 8 weeks | Degrees |  |  |  |  |
|  |  |  |  |  |  |  | Satisfaction with the healthcare provided | (0 to 100) |  |  |  |  |
|  |  |  |  |  | Early active-assisted mobilisation (after two weeks) after cemented hemiarthroplasty | Late mobilisation (after six weeks) after cemented hemiarthroplasty | Shoulder function at 1 year | Oxford Shoulder Score/ Constant score |  | 1 (59) |  |  |
|  |  |  |  |  |  |  | ROM | Degrees in elevation and external rotation at 1 year |  |  |  |  |

Abbreviations: POP: plaster of Paris cast; ROM: range of motion; NR: not reported; CRPS type 1: complex regional pain syndrome type 1; PEMF: pulsed electromagnetic field; PRWE: Patient-Rated Wrist Evaluation; QuickDASH: Disabilities of the Arm, Shoulder and Hand Quick Questionnaire; VAS: Visual Analogue Scale; CPM: continuous passive motion; MCP: metacarpal phalange; MAM-36: Manual Ability Measure-36; MEM: Manual Edema Mobilization; SF-36: Short Form Health Survey 36; EQ-5D: EuroQoL-5 dimensions.
